# Alterations of Resting-State Locus Coeruleus Functional Connectivity After Transdermal Trigeminal Electrical Neuromodulation in Insomnia

**DOI:** 10.3389/fpsyt.2022.875227

**Published:** 2022-05-10

**Authors:** Yoo Hyun Um, Sheng-Min Wang, Dong Woo Kang, Nak-Young Kim, Hyun Kook Lim

**Affiliations:** ^1^Department of Psychiatry, St. Vincent's Hospital, College of Medicine, The Catholic University of Korea, Seoul, South Korea; ^2^Department of Psychiatry, Yeouido St. Mary's Hospital, College of Medicine, The Catholic University of Korea, Seoul, South Korea; ^3^Department of Psychiatry, Seoul St. Mary's Hospital, College of Medicine, The Catholic University of Korea, Seoul, South Korea; ^4^Department of Psychiatry, Keyo Hospital, Keyo Medical Foundation, Uiwang, South Korea

**Keywords:** locus coeruleus, norepinephrine, trigeminal nerve, insomnia, intervention

## Abstract

**Background:**

Transdermal trigeminal electrical neuromodulation (TTEN) is a novel treatment modality that is known for noradrenergic modulation through the trigeminal nerve and locus coeruleus (LC). This study aimed to demonstrate the alterations of LC functional connectivity (FC) in patients with insomnia after a 4-week TTEN.

**Methods:**

The Cefaly device targeting the ophthalmic division of the trigeminal nerve was applied to a total of 12 patients with insomnia to monitor for the effects of TTEN. All the patients went through a 4-week daily 20 min TTEN sessions before bedtime. Baseline and post-TTEN demographic data, polysomnography (PSG) parameters, and insomnia severity index (ISI) were attained. Data from pre- and post-intervention resting-state functional magnetic resonance imaging (MRI) were collected. LC FC differences were measured between the pre-and post-TTEN groups through seed-to-voxel analysis. Correlation analyses were conducted between LC FC changes after TTEN, ISI score changes, and PSG parameter changes.

**Results:**

There was a significantly decreased LC FC with occipital and temporal cortices after a 4-week TTEN. However, there was no significant correlation between LC FC, ISI score changes, and PSG parameter changes.

**Conclusion:**

By targeting hyperarousal symptoms of insomnia, TTEN can be a promising intervention that can modulate LC FC in patients with insomnia patients. The data presented in the study are from a study exploring the effect of TTEN on insomnia (www.clinicaltrials.gov, NCT04838067).

## Introduction

Among many perspectives to understand the etiopathogenesis of insomnia, the hyperarousal model of insomnia has been extensively studied and researched. The hyperarousal model encompasses the concept that subjects with insomnia exhibit autonomic hyperactivity and cortical activation manifested by various biological parameters (1). One of them is the dysregulation of the noradrenergic system, which plays a pivotal role in the modulation of arousal, stress, and anxiety (2). Indeed, autonomic activity was disrupted in older adults with insomnia in one study, with decreased 24-h norepinephrine (NE) levels, and this interacted with slow oscillatory activity in sleep cycles (3). In another study, nocturnal NE levels were increased in insomniacs (4), suggesting autonomic dysregulation mediated by the noradrenergic system may be an important culprit in insomnia.

Norepinephrine cell groups are located in the locus coeruleus (LC), one of many brainstem nuclei involved in sleep-wake regulation. With recent advances in neuroimaging studies, the structural and functional correlates of this small blue nucleus have been gaining increasing attention (5–8). Considering the critical role of the LC-NE system in the induction of hyperarousal in insomnia, neuroimaging studies on the association between the LC and insomnia are scarce. There was a recent study on the LC functional connectivity (FC) in insomnia subjects where LC FC alterations were observed and they were associated with anxiety symptoms in chronic insomnia subjects (9). However, the aforementioned study did not rule out other sleep disorders that can induce chronic insomnia symptoms.

Transdermal trigeminal electrical modulation (TTEN) is a non-invasive stimulation method that targets the ophthalmic division of the trigeminal nerve, and it was reported to produce a sedative effect that resulted in a reduction of psychomotor vigilance (10). This sedative effect was discussed to be a result of modulation of the LC, which results in the deactivation of NE and the resultant reduction in autonomic activity (11, 12). We have previously identified the improvement of subjective parameters and a trend for improved sleep maintenance after a month of TTEN in insomniac subjects (13). However, the exact mechanisms of TTEN on insomniac subjects are unknown.

Previous non-pharmacological interventions on insomniac subjects and their impact on neuroimaging parameters have mainly focused on the impact of cognitive-behavioral therapy for insomnia (CBT-i) on resting-state brain FC. Patients with insomnia demonstrated alterations in subcortical resting-state FC after 5 weeks of CBT-i (14). A study on the influence of CBT-i on dialysis patients with insomnia revealed changes in the default-mode network (DMN) and dorsolateral prefrontal cortex (15). A major weakness of the aforementioned studies is that since the exact neural target of the intervention is not known, it is difficult to confirm the results of the FC alterations observed were indeed the actual effects of the intervention or were observed by chance. However, as for TTEN, since it is well-known for its modulation of the trigeminal nerve and its resultant influence on the LC-NE system, we speculated that looking into the impact of TTEN on the LC FC of patients with insomnia will not only help test the hyperarousal model of insomnia but also help unravel the neurobiological mechanisms of patients with insomnia.

Therefore, in this study, we aimed to explore the impact of a 4-week TTEN on the LC FC of patients with insomnia, who were grouped with stringent diagnostic classification. We hypothesized that there will be distinct alterations in the LC FC after a 4-week period of TTEN.

## Materials and Methods

### Participants

The participants of this study were a subset from the clinical trial to prove the effects of a 4-week TTEN on subjective and objective parameters of patients with insomnia, which were previously published and distributed (13). All the subjects in the aforementioned clinical trial went through sleep surveys, polysomnography (PSG), and magnetic resonance imaging (MRI) both pre-TTEN and post-TTEN. Subjects with an age range of 19–64 years, who scored a total of 15 or more in insomnia severity index (ISI), or with a diagnosis of insomnia disorder according to the Diagnostics and Statistical Manual of Mental Disorders (5th edition) (16), were eligible to participate in the study. Those with an unstable medical condition, a prior diagnosis of sleep disorders, current prescription of hypnotics, such as sedating antidepressants, z-drugs, and benzodiazepines, cognitive impairment, psychiatric disorders, or neurological disorders, a history of brain or facial trauma within 3 months, acrylic acid allergy, electromagnetic hypersensitivity, apnea-hypopnea index (AHI) of >15/h in the baseline PSG, and skin abrasions were excluded from the study. All patients were provided with an option to withdraw from the study and choose other treatments, such as CBT-i or counseling throughout the study period. Out of all the patients who completed the study protocol, a subset of patients was selected to test the hypothesis for the current study. They were identified as having both intact pre-TTEN and post-TTEN resting-state functional MRI results, and they had AHI of <15/h in both pre-TTEN and post-TTEN. Informed consents were received from all participants. All study procedures were conducted with the approval of the Institutional Review Board (IRB) of the St. Vincent's Hospital, the Catholic University of Korea (VC18DNSI0145). Moreover, this clinical trial was approved by the Korean Ministry of Food and Drug Safety (MFDS).

### Measures

#### Insomnia Severity Index (ISI)

The ISI is a self-related scale prevalently used to measure insomnia severity. It consists of seven items scored on a 0–4 scale, and a score of 15 or more indicates insomnia of moderate to severe severity (17). The Korean version of ISI was previously validated by Cho et al. (18). The patients were asked to complete the questionnaire before the application of the Cefaly device and after the 4-week treatment, to determine their eligibility for the study.

#### Transdermal Trigeminal Electrical Neuromodulation (TTEN)

All patients were distributed with the Cefaly device, which was originally approved by the Korean MFDS for the treatment of migraine. An official approval by the Korean MFD was attained to initiate a clinical trial testing the effectiveness of the Cefaly device in patients with insomnia. The Cefaly device delivers electrical micro-impulses through a self-adhesive electrode, which is placed over the participant's forehead to target the supratrochlear and supraorbital branches of the ophthalmic division of the trigeminal nerve (19). The impulse with a width of 250 μs, frequency of 60 Hz, and the maximum intensity of 16 mA was delivered over 14 min (20). All the patients were thoroughly educated on the daily 20-min bedtime usage of the device for 4 weeks. A weekly telemonitoring was implemented to check the compliance of patients.

#### Polysomnography (PSG)

Every participant underwent PSG at night using the EMBLA® S7000 System (Embla Systems, Inc., Broomfield, CO, USA) and Somnologica version 3.3.1. Sleep stage scoring was implemented with the data attained from an electroencephalogram, electrooculogram, and electromyogram for the chin and limbs. Chest and abdomen band transducers were applied and respiratory inductance plethysmography was used to monitor for respiratory effort and ventilation. Airflow was monitored with a nasal pressure sensor and a thermistor. A pulse oximeter was applied to measure arterial blood oxygen saturation. All the sleep parameters were scored and assessed with the American Academy of Sleep Medicine manual for the scoring of sleep (21) both pre-TTEN and post-TTEN.

#### MRI Acquisition

Imaging data were collected at the Department of Radiology, St Vincent's Hospital, The Catholic University of Korea, using a 3T Siemens Verio machine and an eight-channel Siemens head coil (Siemens Medical Solutions, Erlangen, Germany). The parameters used for the T1-weighted volumetric magnetization-prepared rapid gradient echo scan sequences were echo time (TE) = 2.5 ms, repetition time (TR) = 1,900 ms, an inversion time = 900 ms, field-of-view (FOV) = 250 mm, matrix = 256 × 256, voxel size = 1.0 mm × 1.0 mm × 1.0 mm, and 160 slices. Resting-state functional images were collected using a T2^*^ weighting gradient echo sequence with TR = 2,490 ms, TE = 30 ms, matrix = 128 × 128 × 29, and voxel size = 2 mm × 2 mm × 3 mm. Briefly, 150 volumes were acquired over 5 min with the instruction “keep your eyes closed and think of nothing in particular.”

### Resting-State FC Analysis

#### Functional Pre-processing

The CONN Toolbox Version 20.b. was utilized to preprocess and analyze functional imaging data and calculate the FC of the LC (22). The default CONN preprocessing pipeline was utilized, with a sequential process of realignment, unwarping, slice-time correction, scrubbing with Artifact Detection and Removal Tool (ART)-based identification for outlier scans, segmentation into gray matter, white matter, and cerebrospinal fluid (CSF), normalization to the Montreal Neurological Institute (MNI) template, smoothing using an 8-mm Gaussian kernel. After preprocessing, the CONN denoising pipeline was implemented to remove possible confounders in the BOLD signal. Through the adoption of linear regression and band-pass filter of [0.008 0.09] Hz, removal of white matter, CSF noise components, unwanted subject motion, and physiological noises were conducted.

#### Definition of LC Seed

We used an LC mask which was previously created by Keren et al., the probabilistic map of LC containing LC peak signal coordinates at two standard deviations (SDs) (5).

#### Seed-to-Voxel Analysis

In first-level analyses, computation of seed-to-voxel connectivity maps was implemented in each subject, and these measures were adopted in group-level analysis. Seed-to-voxel analysis was performed using pre- and post-TTEN contrasts to determine whether there are statistically significant differences in the LC FC between pre- and post-TTEN scans. All comparisons throughout the whole brain adopted voxel-wise statistics, threshold at *p* < 0.05, false discovery rate (FDR) corrected for cluster level, and *p* < 0.001, uncorrected for voxel level. The analyses were performed in Matlab 2021b (The Mathworks, Inc.).

### Statistical Analysis

First, the baseline demographic data were presented with descriptive statistics. The pre- and post-TTEN ISI and PSG parameters [total sleep time (TST), wake after sleep onset (WASO), sleep efficiency (SE), sleep latency (SL), rapid eye movement sleep latency (REML), N1 % of total sleep time(N1), N2 % of total sleep time (N2), N3 % of total sleep time (N3), REM % of total sleep time(REM)] were compared with a paired *t*-test. Additionally, correlations of ΔISI (pre-TTEN ISI score subtracted from post-TTEN ISI score), ΔTST (pre-TTEN TST subtracted from post-TTEN TST), ΔWASO (pre-TTEN WASO subtracted from post-TTEN WASO), ΔSE(pre-TTEN SE subtracted from post TTEN), ΔSL(pre-TTEN SL subtracted from post-TTEN SL), ΔREML(pre-TTEN REML subtracted from post-TTEN REML), ΔN1(pre-TTEN N1 subtracted from post-TTEN N1), ΔN2(pre-TTEN N2 subtracted from post-TTEN N2), ΔN3(pre-TTEN N3 subtracted from post-TTEN N3), ΔREM (pre-TTEN REM subtracted from post-TTEN REM), and the ΔFC z-score (pre-TTEN z score of the FC map subtracted from post-TTEN z score of the FC map) were explored with Pearson's correlation analysis. Data were analyzed using R software (www.r-project.org).

## Results

A total of 12 patients participated in the study. Detailed demographic data of the participants are summarized in Table 1. The mean age of the participants was 42.83 ± 8.38 years, and the mean education years were 13.83 ± 2.59. The mean insomnia severity index of the participants was 20.50 ± 4.32. Baseline PSG parameters demonstrated that the mean wake after sleep onset of the participants was increased to 42.44 ± 28.73 min. Mean sleep efficiency and sleep onset latency of the participants were not markedly affected, showing 85.92 ± 8.50% and 26.23 ± 20.33 min, respectively. The mean REM latency was 89.68 ± 43.24 min, and the mean N1, N2, N3, and REM sleep proportions of total sleep were 11.89 ± 3.86%, 54.87 ± 7.88%, 6.32 ± 7.94%, 33.26 ± 36.79%, respectively. The mean apnea-hypopnea index of the participants was 7.34 ± 4.42/h.

**Table 1:** Demographic characteristics and baseline polysomnography (PSG) parameters of participants (*N* = 12).

**Demographic characteristics**	
Age (mean ± SD)	42.83 ± 8.38
Sex (M:F)	3:9
Education years (mean ± SD)	13.83 ± 2.59
Insomnia severity index (mean ± SD)	20.50 ± 4.32
Baseline polysomnography parameters	mean ± SD
Total sleep time (minutes)	419.16 ± 43.48
Wake after sleep onset (minutes)	42.44 ± 28.73
Sleep efficiency (%)	85.92 ± 8.50
Sleep onset latency (minutes)	26.23 ± 20.33
REM latency (minutes)	89.68 ± 43.24
N1 of total sleep time (%)	11.89 ± 3.86
N2 of total sleep time (%)	54.87 ± 7.88
N3 of total sleep time (%)	6.32 ± 7.94
REM of total sleep time (%)	33.26 ± 36.79
AHI (/hour)	7.34 ± 4.42

*SD: standard deviation*.

After a month of TTEN, post-TTEN ISI score (9.92 ± 5.76) was significantly improved when compared with pre-TTEN (20.50 ± 4.32) (*p* < 0.001). Moreover, there was a significant reduction in WASO after a month of TTEN, from 42.44 ± 28.73 to 30.43 ± 16.18 min (*p* = 0.046). Paired *t*-test results on the effects of a 4-week TTEN on the ISI and objective parameters of PSG are presented in Supplementary Table 1.

**Figure 1:**
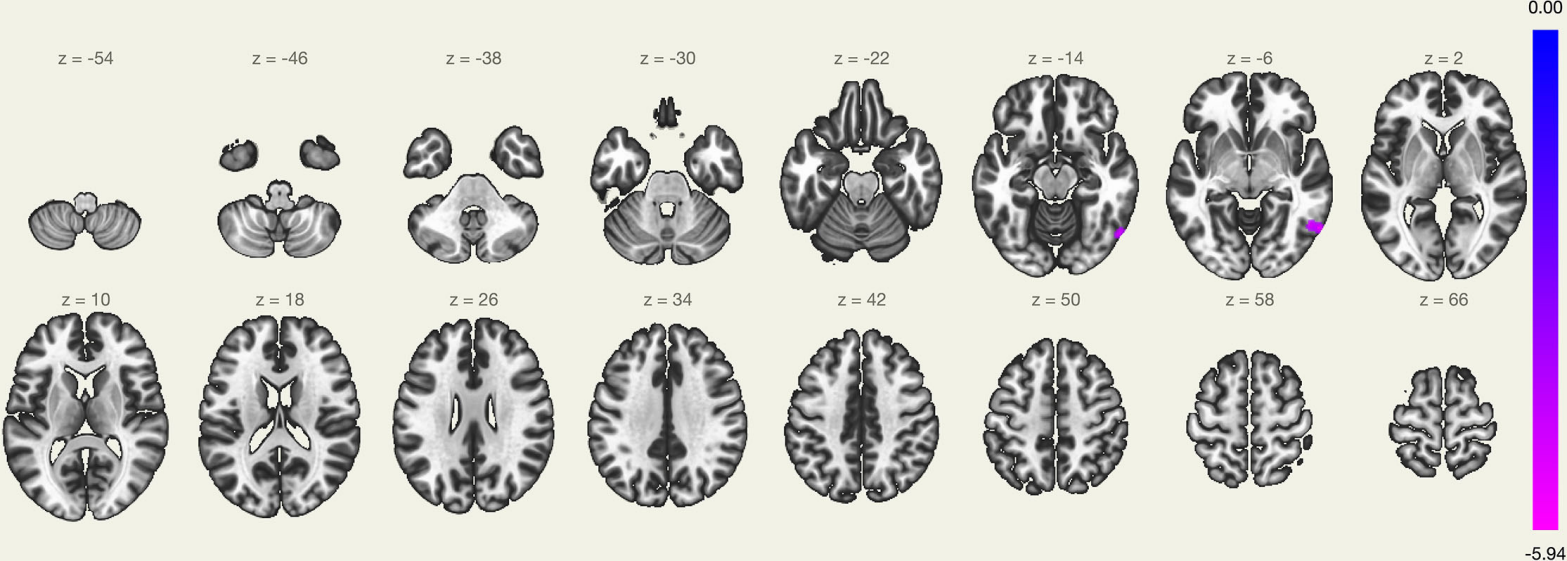
Regions showing different functional connectivity with the locus coeruleus (LC) in the pre-transdermal trigeminal electrical neuromodulation (TTEN) and post-TTEN group.

The LC FC with the right lateral occipital cortex, right inferior temporal gyrus, and right middle temporal gyrus (+60, −60, and −04) was significantly decreased (Table 2 and Figure 1). There was no significant correlation noted between ΔISI score, ΔTST, ΔWASO, ΔSE, ΔSL, ΔREML, ΔN1, ΔN2, ΔN3, ΔREM, and ΔFC z-score (Table 3).

**Table 2:** Regions showing different locus coeruleus (LC) resting functional connectivity (FC) between pre-TTEN and post-TTEN (between-group analysis, voxel threshold: *p* < 0.001, uncorrected, cluster threshold: *p* < 0.05, and cluster-size p-FDR corrected).

**Seed region & network**	**Clusters (x,y,z)**	**Regions covered by the cluster**	**Cluster size**	**Size p-FDR**	**Peak p-uncorrected**
**Post-TTEN < Pre-TTEN**					
**Locus coeruleus**	+60,−60,−04	1) Right lateral occipital cortex, inferior division 2) Right inferior temporal gyrus, temporooccipital part 3) Right middle temporal gyrus, temporooccipital part	228	0.000045	0.000006

*TTEN, transdermal trigeminal electrical neuromodulation, FDR, false-discovery rate*.

**Table 3:** Correlation between LC FC changes and clinical parameters.

		**ΔISI**	**ΔTST**	**ΔWASO**	**ΔSE**	**ΔSL**	**ΔREML**	**ΔN1**	**ΔN2**	**ΔN3**	**ΔREM**
ΔFC	Pearson's r	−0.308	−0.386	0.104	−0.309	0.448	−0.211	0.007	0.104	−0.348	0.540
	P value	0.330	0.216	0.747	0.328	0.144	0.511	0.982	0.747	0.268	0.070

*LC, locus coeruleus; FC, functional connectivity; ISI, insomnia severity index; TST, total sleep time; WASO, wake after sleep onset; SE, sleep efficiency; SL, sleep latency; REML, rapid eye movement sleep latency; N1, N1(%) of total sleep time; N2, N2(%) of total sleep time; N3, N3(%) of total sleep time; REM, REM(%) of total sleep time*.

## Discussion

To the best of our knowledge, this is the first study to look into the impact of TTEN on the LC FC of patients with insomnia. After a month of TTEN intervention, the LC FC with the occipital and temporal cortices were significantly decreased. These findings convey several important clinical implications.

First, the LC FC with right lateral occipital was significantly decreased in our study subjects after a month of TTEN. A recent study by Gong et al. reported increased LC FC with left supramarginal gyrus and left middle occipital gyrus in patients with chronic insomnia disorder (9). Additionally, a recent systematic review indicated that FC increments in visual pathways were observed in insomnia disorder (23). A month of TTEN may have reversed this process, by decreasing LC FC with the occipital cortex. Our results are consistent with a previous study on patients with insomnia, in which right medial prefrontal cortex FC with occipital cortex was reduced after transcutaneous auricular vagus nerve stimulation (24). Considering alterations of visual cortex FC in patients with insomnia frequently observed in previous studies, the association between the LC-NE system, occipital cortex, and insomnia should not be overlooked. Indeed, the LC-NE activations can intricately modulate occipital cortex activity, enhancing some signals while depressing others in a dose-dependent manner (25). Increased LC-FC with occipital cortex may be an important biomarker of insomnia disorder, and this phenomenon may be a byproduct of increased sensitivity to the ‘signal’ related to arousal mediated by the LC-NE system (26). Moreover, impairments in the tonic and phasic firing may have resulted in a high tonic and low phasic state of the LC-NE system, which may have resulted in a hyperarousal state in patients with insomnia (26). TTEN, by a modulation of the LC-NE system, may have reversed this phenomenon.

Secondly, the LC FC with right and middle temporal cortices was significantly decreased after TTEN.

Previous studies have endorsed that the dorsolateral prefrontal cortex and middle temporal cortex were two major hub nodes of insomnia, where decreased FC was observed (23, 27). No studies to date have elucidated the relationship of the LC FC with temporal cortices in patients with insomnia. In another study, patients with insomnia disorder showed reduced amplitude of low-frequency fluctuations in bilateral middle temporal cortices (28). On the other hand, a study on the graph theoretical analysis of network hubs in primary patients with insomnia reported increased FC between the right middle temporal gyrus with the executive control network, interpreting it as a representation of the reduced ability to disconnect from external auditory stimuli often observed in patients with insomnia (29). A recent study on a rat model demonstrated that the LC-NE system is a determinant of sensory-evoked awakenings from sleep (30). Deceased LC FC with right and middle temporal cortices after TTEN may represent reduced arousal-related activation of the LC-NE system and decreased sensitivity to external stimuli, which may help ameliorate hyperarousal in patients with insomnia.

However, there was no significant correlation between changes in subjective, objective sleep parameters, and ΔFC z-score in the present study. This may be due to our small sample size, and more importantly, the LC FC changes in response to TTEN may have represented a mediating influence in ameliorating insomnia symptoms, but not a direct effect. The LC FC changes may have correlated with the hyperarousal level of our participants, which we did not measure in the current study. Adopting measures to evaluate and quantify the hyperarousal level of patients with insomnia in futures studies in exploring the correlation with the LC FC will be conducive to understand the role of LC FC changes in hyperarousal and insomnia.

There are several limitations to the study that must be taken into consideration. First, the sample size was small, thus limiting the generalizability of our results. Second, since our participants went through TTEN only for a month, the resting-state LC FC changes observed represent the acute effects of TTEN. Third, our study was not sham-controlled. However, there are reports of huddles in applying a genuine sham device and high drop-out rates in neurostimulation studies (31). Fourth, we did not measure anxiety levels in our subjects, which may have contributed to the hyperarousal state of the participants and which may have acted as a potential confounding factor. Last, the timing of MRI acquisition may have confounded the results, whether it was taken in the morning, afternoon, or evening since the hyperarousal level of an individual can differ during the day.

In conclusion, our study confirms the LC FC alterations in patients with insomnia and the modulatory effect of TTEN on LC FC in insomnia. Despite the high prevalence of insomnia in clinical settings, biomarkers of insomnia, classification of insomnia phenotypes, and exact neurobiological mechanisms of insomnia are still not confirmatory. Unraveling the neural correlates of insomnia, targeting the disrupted brain regions in insomnia treatment, and confirming the treatment response are major questions that remain elusive. A recent study demonstrated a potential neurostimulation target in insomnia, using resting-state FC maps (32). These attempts should be continuously made, and we believe that our results will be of help in establishing the basis for the aforementioned queries in the diagnosis and treatment of insomnia.

## Data Availability Statement

All relevant data is contained within the article: The original contributions presented in the study are included in the article/Supplementary Material, further inquires can be directed to the corresponding author.

## Ethics Statement

The studies involving human participants were reviewed and approved by the Institutional Review Board (IRB) of the St. Vincent's Hospital, the Catholic University of Korea. The patients/participants provided their written informed consent to participate in this study.

## Author Contributions

YU: data collection, data analysis and interpretation, and drafting the article. S-MW: critical revision of the article and final approval of the version to be published. DK: critical revision of the article and final approval of the version to be published. N-YK: critical revision of the article and final approval of the version to be published. HL: conception, design of the work, critical revision of the article, and final approval of the version to be published. All authors contributed to the article and approved the submitted version.

## Funding

This was not an industry supported study. This work was supported by the National Research Foundation of Korea (NRF) grant funded by the Korea government (MSIT) (Nos. 2018R1D1A1A02049615 and 2020R1I1A1A01057792).

## Conflict of Interest

The authors declare that the research was conducted in the absence of any commercial or financial relationships that could be construed as a potential conflict of interest.

## Publisher's Note

All claims expressed in this article are solely those of the authors and do not necessarily represent those of their affiliated organizations, or those of the publisher, the editors and the reviewers. Any product that may be evaluated in this article, or claim that may be made by its manufacturer, is not guaranteed or endorsed by the publisher.
