# Supplementary material for: Alterations of Resting-State Locus Coeruleus Functional Connectivity After Transdermal Trigeminal Electrical Neuromodulation in Insomnia
Source: Front Psychiatry. 2022 May 10;13:875227. doi: 10.3389/fpsyt.2022.875227 (PMC9127056; doi:10.3389/fpsyt.2022.875227)
Supplement: Supplementary file 1 [file Table_1.DOCX]

**Supplementary Table1. Effects of 4-week TTEN on subjective and objective sleep parameters in insomnia patients**

|  | Pre-TTEN | Post-TTEN | P value |
| --- | --- | --- | --- |
| Subjective parameters | | | |
| Insomnia severity index | 20.50±4.32 | 9.92±5.76 | <0.001 |
| Objective parameters | | | |
| Total sleep time (min) | 419.17±43.48 | 424.38±30.57 | 0.665 |
| Wake after sleep onset(min) | 42.44±28.73 | 30.43±16.18 | 0.046 |
| Sleep efficiency (%) | 85.92±8.50 | 89.03±5.60 | 0.145 |
| Sleep latency(min) | 26.23±20.33 | 22.72±17.23 | 0.513 |
| REM latency(min) | 89.67±43.24 | 93.79±47.65 | 0.680 |
| N1 of total sleep time (%) | 11.89±3.86 | 11.05±3.08 | 0.399 |
| N2 of total sleep time (%) | 54.87±7.88 | 58.48±5.26 | 0.058 |
| N3 of total sleep time (%) | 6.32±7.94 | 6.08±5.63 | 0.836 |
| REM of total sleep time (%) | 33.27±36.79 | 19.36±5.01 | 0.252 |

Presented as Mean±SD, REM, rapid eye movement, TTEN, transdermal trigeminal electrical neuromodulation, SD, standard deviation
